# Spatial analysis of a hydrocarbon waste‐remediating landfarm demonstrates influence of management practices on bacterial and fungal community structure

**DOI:** 10.1111/1751-7915.13397

**Published:** 2019-03-29

**Authors:** Jordyn Bergsveinson, Benjamin J. Perry, Gavin L. Simpson, Christopher K. Yost, Robert J. Schutzman, Britt D. Hall, Andrew D. S. Cameron

**Affiliations:** ^1^ Department of Biology University of Regina Regina SK Canada; ^2^ Institute for Microbial Systems and Society University of Regina Regina SK Canada; ^3^ Institute of Environmental Change and Society University of Regina Regina SK Canada; ^4^ EVRAZ Inc. NA Canada Regina SK Canada; ^5^ Present address: Department of Microbiology and Immunology University of Otago Dunedin New Zealand

## Abstract

Cultivation of dedicated soil plots called ‘landfarms' is an effective technology for bioremediation of hydrocarbon waste generated by various industrial practices. To understand the influence of soil conditions on landfarm microbial communities, analysis of bacterial and fungal community structure using next‐generation sequencing at different sections and depths was performed across a hydrocarbon‐waste landfarm in Regina, Saskatchewan, Canada. While a core set of hydrocarbon‐associated bacterial and fungal taxa are present throughout the landfarm, unique bacterial and fungal operational taxonomic units are differentially abundant at sections within the landfarm, which correlate with differences in soil physiochemical properties and management practices. Increased frequency of waste application resulted in strong positive correlations between bacterial community assemblages and elevated amounts of oil, grease and F3 – F4 hydrocarbon fractions. In areas of standing water and lower application of hydrocarbon, microbial community structure correlated with soil pH, trace nutrients and metals. Overall, diversity and structure of bacterial communities remain relatively stable across the landfarm, while in contrast, fungal community structure appears more responsive to soil oxygen conditions. Results are consistent with the hypothesis that years of bioremediation activity have shaped microbial communities; however, several management practices can be undertaken to increase efficiency of remediation, including the removal of standing water and soil tilling across the landfarm.

## Introduction

Soils naturally contain diverse microorganisms capable of degrading complex hydrocarbons arising from industrial activity. Controlled application of industrial waste to topsoil has provided reliable bioremediation to reduce hydrocarbon waste in various industries (Khan *et al*., 2004). These treatment plots are referred to as ‘landfarms', and the development of best practices for the maintenance of these systems for *ex‐situ* treatment of wastes is attractive as a cost‐saving and environmentally responsible form of waste disposal (Rhykerd *et al*., 1999; Besalatpour *et al*., 2011). Like other bioremediation technologies, treatment efficacy of landfarms depends on multiple physical, chemical and biological features, including land area, soil aeration, nutrient availability, pollutant mobility and toxicity, and microbial biodiversity (Rhykerd *et al*., 1999; Straube *et al*., 2003; Khan *et al*., 2004; Harmsen *et al*., 2006).

Effective landfarm management practices will consider and modulate these soil features to improve the activity of the bacterial and fungal communities, which are the major drivers of the biological degradation of hydrocarbons and other waste compounds applied to landfarms and other remediation technologies (Leahy *et al*., 2003; Straube *et al*., 2003; Seo *et al*., 2009; Militon *et al*., 2010). These studies focused on the effects of operational parameters such as tillage, waste dosing/application rates and moisture content on overall remediation efficacy, but did not consider the effects of management practices on microbial community structure and variability. Alternatively, several studies have examined the structure of bacterial communities that remediate specific pollutants, either using terminal restriction fragment length polymorphism (TRFLP) analysis of 16S rRNA genes present in a petroleum refinery landfarm (Katsivela *et al*., 2005) or denaturing gradient gel electrophoresis (DGGE) analysis of 16S rRNA genes to investigate the bacterial community in a lubricant and diesel oil‐polluted landfarm microcosm (Wang *et al*., 2016). While these studies have contributed to understanding the nature of landfarm bacterial communities, no study has yet to perform extensive or high‐resolution analysis of both bacterial and fungal communities throughout an operational landfarm.

Here we apply high‐resolution next‐generation sequencing to profile the bacterial and fungal taxa of a long‐term operational landfarm in Regina, Saskatchewan, Canada, and analyse community structure and diversity in response to hydrocarbon application, soil depth and soil saturation. Such an established landfarm is expected to contain mature microbial communities selected for their ability to metabolize complex hydrocarbons, and thus presents an ideal opportunity to characterize the response and stability of microbial communities to landfarm management activities.

## Results

### Landfarm soil characteristics

**Figure 1:**
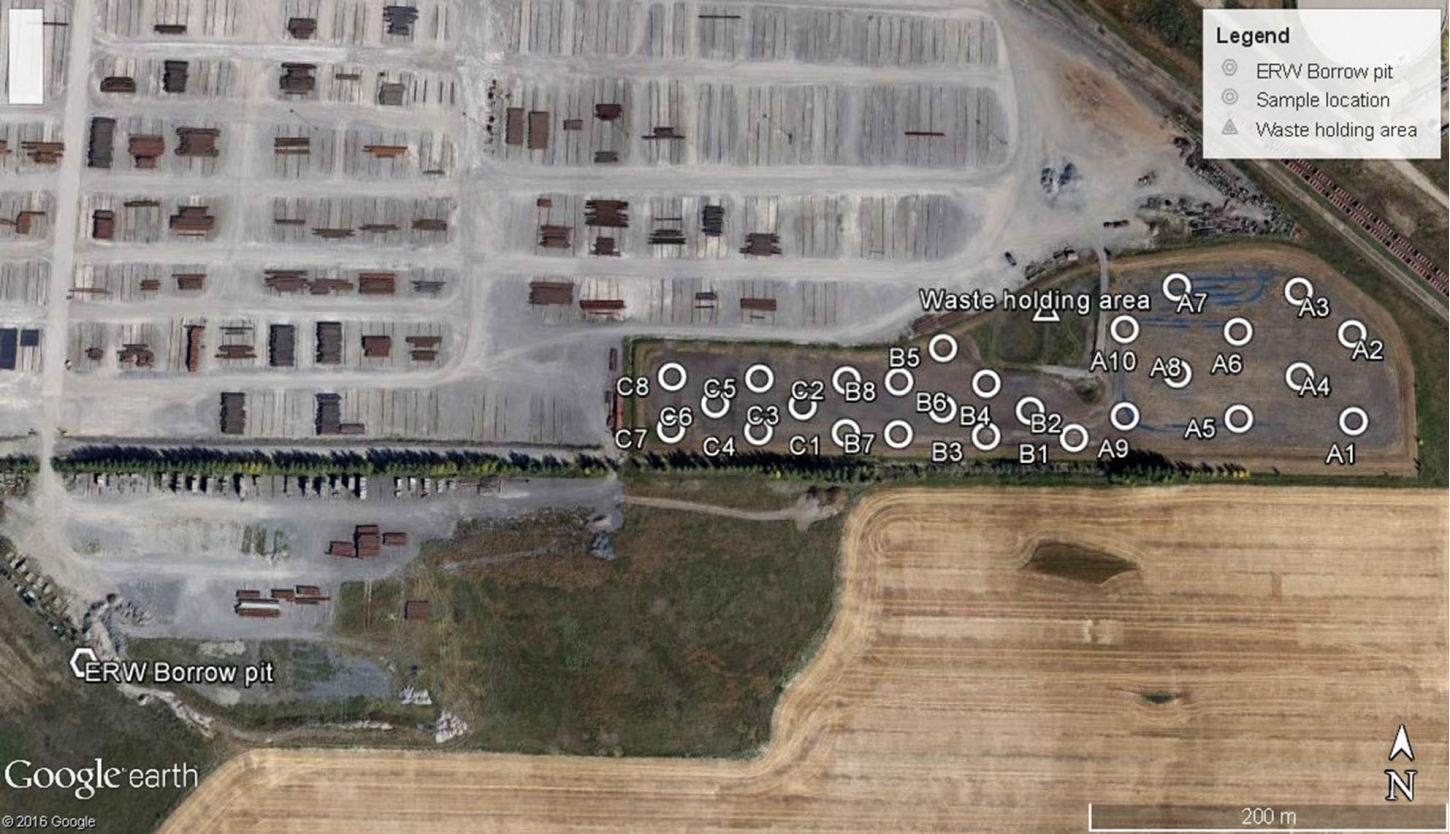
Sample locations within the EVRAZ landfarm. Soil samples were collected from ten sites within A, B and C sections of the landfarm, and from an excavation site (ERW; Control) 500 m southeast of the landfarm. Map data attributed to Google Earth and DigitalGlobe ©2016.

**Figure 2:**
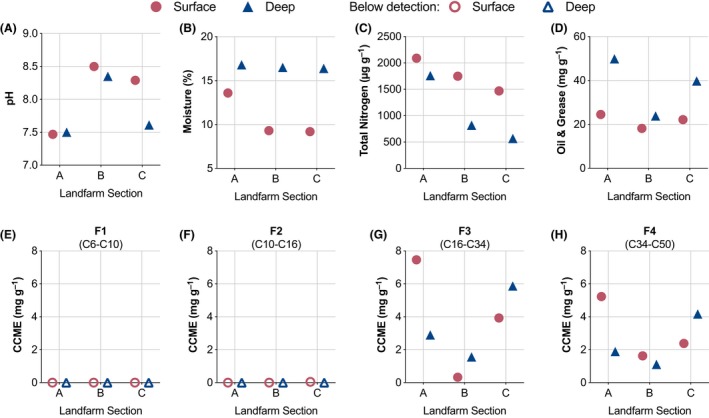
Soil chemistry and moisture. A selection of soil characteristics provided in Table S1 are presented in graphical format for landfarm sections A, B and C, in surface and deep sampling depths collected in June. Raw and additional data are included in Table S1.

The EVRAZ Inc. landfarm is divided into three separate management sections, A, B and C surrounding a waste holding area (Fig. 1), with the collected hydrocarbon waste being applied in late spring (Table 1). Soil samples were collected at two depths, ‘surface' (0–15 cm) and ‘deep' (15–30 cm) from ten sites of landfarm sections (and once from nearby borrow‐pit, ERW; Fig. 1), following tilling of the site in June 2015, with ‘surface' samples collected again in November (Table 1). Multiple features of soil chemistry are routinely measured as part of landfarm management, providing critical insights into the abiotic features of the landfarm soils. Section A is characterized by features important for robust biological activity: a near neutral pH, the highest moisture and the highest concentrations of nitrogen and carbon (Fig. 2). Petroleum‐related hydrocarbons BTEX plus the Canadian Council of Ministers of the Environment (CCME) hydrocarbon fractions F1 (number of carbons ranging from: C6–C10) and F2 (C10–C16) were below detection limits across all sites, except for trace amounts of F2 hydrocarbons detected in section C surface soils (Fig. 2). The long CCME carbon chain F3 (C16–C34) and F4 (C34–C50) fractions are more recalcitrant hydrocarbons and include polycyclic aromatic hydrocarbons. Both landfarm sections A and C received fresh applications of hydrocarbon‐contaminated clays in April 2015, 2 months before sampling in June 2015 (Table 1), contributing to the high concentrations of fractions F3 and F4 in sections A and C. In section A, total carbon, oil and grease (which includes non‐water‐soluble oils and greases), and the CCME hydrocarbon fractions were most abundant in surface soils, whereas section C demonstrated the converse pattern of higher hydrocarbon levels in deep soils (Fig. 2).

**Table 1:** Timeline of events at landfarm between April and November 2015

Date	Event	Details	Location^a^
6–15 April	Application of waste	Hydrocarbon‐impacted clay	Section A
20 April	Application of waste	Hydrocarbon‐impacted clay	Section C
5 June	Tilling	Landfarm soil turned using a smooth blade grader at a depth of 10 cm	Entire landfarm
9 June	Application of waste	Hydrocarbon‐impacted clay	Section A
10 June	Samples collected for biological and chemical analysis	Surface 0–15 cm; Deep 15–30 cm	26 stations
23 October	Samples collected for biological analysis	Sampling of ERW Control site; 0–5 cm	Control
2 November	Application of waste	Diesel, gas, oil, and hydraulic fluid impacted absorbent pads, oil and diesel impacted sand, slag, soil, contaminated ice	Sections A and C
2 November	Fertilization	~2500 kg of 43% NO_3_ (containing urea) was used to apply 94.87 to A, 35.11 to B, and 59.52 kg to C	Entire landfarm
20 November	Samples collected for biological analysis	0–15 cm	24 stations

**a**. Refer to Fig. 1.

### Landfarm microbial diversity

To survey microbial diversity, the 16S rRNA gene (bacterial) and ITS‐2 region (fungal) taxonomic markers were sequenced for all soil samples. Sequencing error rate was 0.002%, as determined by the mock community, and Goode's coverage estimates, which are the proportion of DNA sequences belonging to operational taxonomic units (OTUs) that have been observed more than once, averaged 0.956 for bacterial and 0.990 for fungal communities, indicating sufficient DNA sequencing coverage to profile microbial communities in all samples. Up to 1200 bacterial and 400 fungal OTUs were identified in each sample, with a total of twenty‐three bacterial and 7 fungal phyla identified in the landfarm.

**Figure 3:**
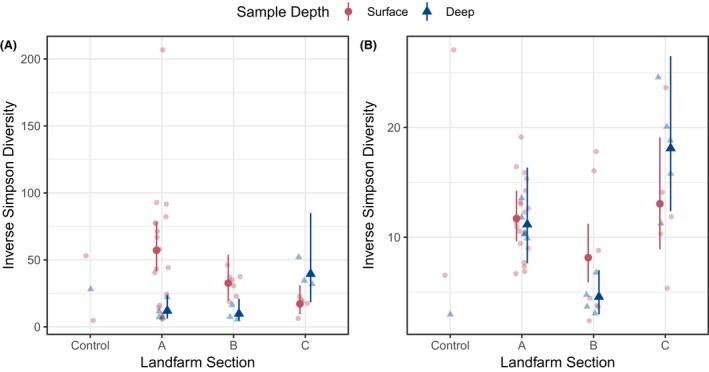
Bacterial (A) and fungal (B) OTU diversity of EVRAZ landfarm soil measured by Inverse Simpson's diversity index. Inverse Simpson's diversity values for all samples (June and November) are plotted (faded points). Solid points are estimated effects from the GLM with bars indicating 95% confidence intervals.

A generalized linear model (GLM) demonstrates that bacterial diversity is significantly higher in surface soil within landfarm sections A (effect estimate −1.557; *P *<* *0.00001) and B (effect estimate ‐1.2044; *P *=* *0.0254) (Table 2), with section A receiving more frequent waste application (Fig. 3). Alternatively, it is expected that the deep section B soils are the most anaerobic within the landfarm, given the soil‐saturating presence of standing water, which corresponds to the statistically modelled difference between the surface and deep samples of section B (Table 2; Fig. 3). Interestingly, there was no significant difference in fungal diversity between surface or deep soils within any of the three landfarm sections (Table 2). However, an effect of standing water on section B fungal communities was observed when we examined differences in diversity between sections by combining surface and deep samples from each section. By this measure, bacterial diversity was not significantly different across the landfarm, but fungal diversity was significantly lower in section B compared to sections A (effect estimate 0.62161; *P *=* *0.00146) and C (effect estimate −0.92973; *P *<* *0.00001) (Table 2; Fig. 3B).

**Table 2:** Simultaneous tests of general linear hypotheses for differences between and effect of landfarm section and depth.^a^

	Null hypotheses^b^	Bacteria			Fungi		
		Effect estimate	*Z* value	Adjusted *P*‐value	Effect estimate	*Z* value	Adjusted *P*‐value
Differences in depth averaged over sections	Surface – Deep = 0	0.5325	2.104	0.121	0.08738	0.567	0.92978
Effect of depth within land section	A_Deep_ – A_Surface_ = 0	−1.5557	−4.240	< 0.0001***	−0.04721	−0.221	0.9947
	B _Deep_ – B_Surface_ = 0	−1.2044	−2.630	0.0254*	−0.57704	−2.162	0.0892
	C _Deep_ – C_Surface_ = 0	0.8326	1.718	0.2361	0.32737	1.215	0.5332
Differences between sections averaged over depths	A – B = 0	0.3031	1.071	0.665	0.62161	3.558	0.00146**
	A – C = 0	−0.1165	−0.389	0.976	−0.30811	−1.715	0.26786
	B – C* *=* *0	−0.4196	−1.302	0.510	−0.92973	−4.783	< 0.0001***

P‐values: * <0.05, ** <0.005, and ***<0.0005.

**a**. Effect of month (June and November) insignificant, and small replicate number of control samples excluded from analysis.

**b**. Null hypotheses tested is no difference between groups (=0). Null hypotheses rejected at level of *P* < 0.05.

### Landfarm microbial taxonomy

**Figure 4:**
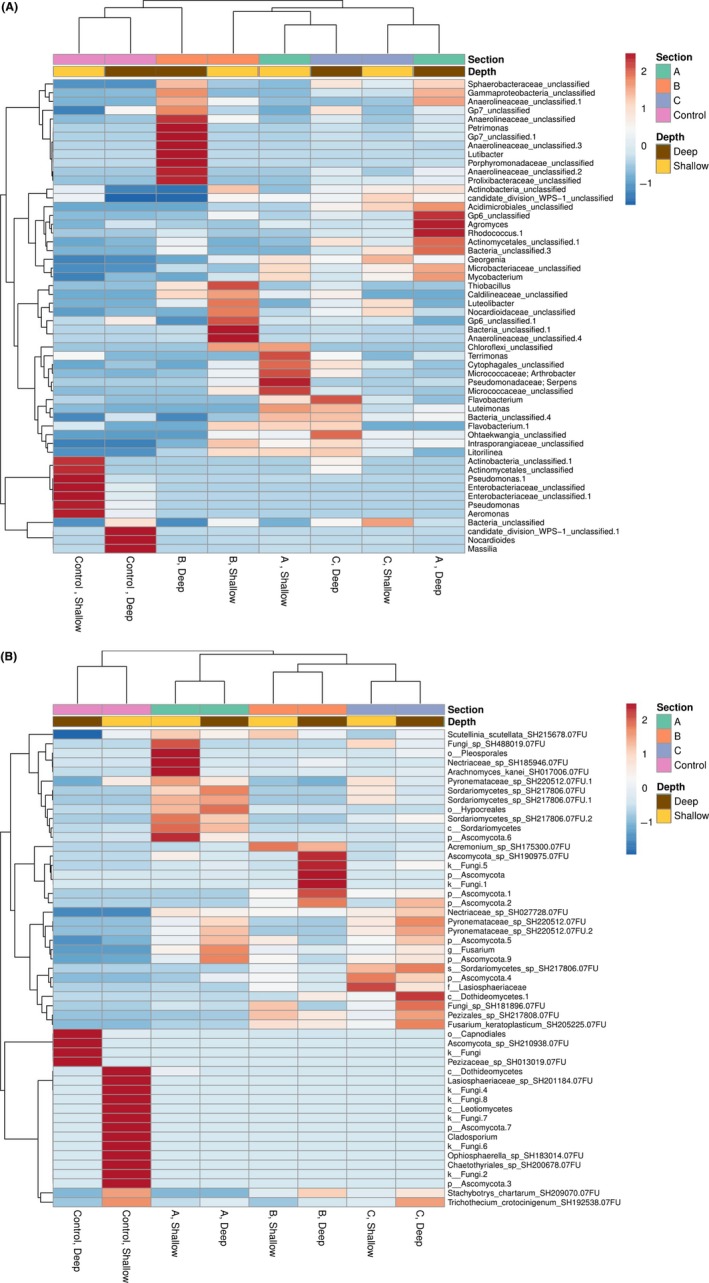
(A) Clustering of top 50 most abundant fungal OTUs. All samples collected from June and November are included and averaged across section and depth. Columns are labelled according to each section and depth, and the median calculated for each OTU in these sample types. OTU abundance values are centred across rows. Heatmap legend indicates result of unit variance scaling, where the standard deviation from a mean (0) is calculated across all columns and can be compared for each OTU between sample type. Both rows and columns are clustered using correlation distance and average linkage. For each fungal OTU in (B), the most refined taxonomic annotation is provided, with p_; c_; o_; f_; g_ indicating phylum, class, order, family and genus, respectively, as few OTUs are able to be identified at the genus level.

Differential abundance analysis of bacterial and fungal OTUs across landfarm sections demonstrate that landfarm sites are distinct from the control ERW site (Fig. 1), which is presumed to represent the natural and ancestral state of the landfarm soils (Fig. 4A and B). When considering the 50 most abundant bacterial OTUs, landfarm sections do not form natural clusters (Fig. 4A), revealing that despite the similarities in overall diversity described above, there is heterogeneity among the most abundant taxa between sections and at both depths within sections. Section B samples contained the most distinct OTU abundance profiles, as section B surface and deep OTUs did not cluster with each other nor closely to the other sections and control soils. Multiple classes of bacteria that metabolize hydrocarbons in the absence of oxygen were found only in the water‐saturated soils of section B. For instance, Anaerolineaceae, which include anaerobic bacteria associated with *n*‐alkane degradation (Liang *et al*., 2016), was abundant and common, particularly in deep soils (Fig. 4A). Although it did not rank in the few most abundant illustrated in Fig. 4A, Hydrogenophilales (*Thiobacillus*), a microaerophilic hydrogen‐oxidizing bacterial group (Orlygsson and Kristjansson, 2014), that was also particularly abundant in section B (Table S2).

Across the landfarm, common soil residents Actinomycetales were the most abundant order of bacteria (Table S2) and soils were highly enriched in taxonomic groups identified in other hydrocarbon‐contaminated soils and water (Peng *et al*., 2015), consistent with the hypothesis that EVRAZ landfarm soils communities have been refined by years of bioremediation activity. These include (but are not limited to) bacterial taxonomic groups of Pseudomonadales (Leahy *et al*., 2003; Seo *et al*., 2009), Acidobacter (Seo *et al*., 2009; Peng *et al*., 2015), Gammaproteobacteria and Actinobacteria (Militon *et al*., 2010; Peng *et al*., 2015), Chloroflexi, Planctomycetes and Bacteroidetes (Peng *et al*., 2015) (Table S2). Specific genera previously demonstrated to be associated with hydrocarbon‐impacted sites and high molecular weight polyaromatic hydrocarbons, such as bacterial OTUs identified as Anaerolineaceae, Caldilineaceae (Zhang *et al*., 2017) and *Lutibacter* (McFarlin *et al*., 2018) are shown to be elevated in abundance in samples from shallow and deep soils of A and B relative to section C (Fig. 4A). Additionally, hydrocarbon‐associated OTUs *Terrimonas* (Adrion *et al*., 2016) and *Agromyces* (Zhang *et al*., 2010) were found abundant in section A soil, while *Thiobacillus* (Militon *et al*., 2010), and *Petrimonas* (Grabowski *et al*., 2005)*,* were found in higher abundances in section B soil.

When fungal OTU abundance is considered, surface and deep samples cluster together within each section, while ERW forms the most divergent cluster (Fig. 4B). Ascomycota were the most abundant order of fungi (Table S3), and fungal hydrocarbon‐related taxonomic orders including Pezizomycetes (Bell *et al*., 2014), Pleosporales (Bourdel *et al*., 2016; Kolařík *et al*., 2017; Mohammadian *et al*., 2017), Hypocreales (Prenafeta‐Boldu *et al*., 2005; Mohammadian *et al*., 2017), Capnodiales (Mohammadian *et al*., 2017), Chaetothyriales (Badali *et al*., 2008) and Tremellales (de Garcia *et al*., 2012) (Table S3). Specific OTUs belonging to *Sordariomycetes* previously demonstrated to be associated with hydrocarbon‐impacted sites and high molecular weight polyaromatic hydrocarbons (Marchand *et al*., 2017) are increased in abundance in deep A and B section soils relative to section C (Fig. 4B).

### Influence of soil parameters on community structure

**Figure 5:**
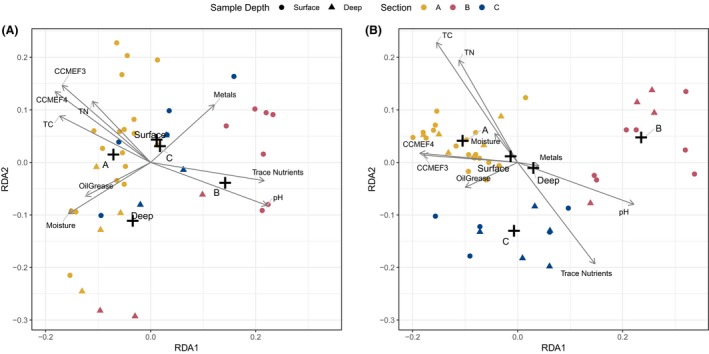
Redundancy analysis of bacterial (A) and fungal (B) OTU abundance and correlation with geochemical parameters. Cross marks represent the fitted centroid ‘mean effect' of the two factors (section and depth) on variation in OTUs. ‘Metals'* *=* *sum of As, Cd, Co, Mn, Mo; ‘Trace Nutrients'* *=* *sum of Ca, K, Na; ‘TC' = Total Carbon; ‘TN' = Total Nitrogen. Arrow length is scaled according to R2 value, thus the length of each vector represents the strength of correlation or magnitude of change in the direction of each arrow.

Redundancy analysis (RDA) provides a powerful approach to examine associations between microbial community structure and soil characteristics. RDA of fungal OTUs showed a tighter clustering of individual samples within sections, and greater spatial distance between sections compared to bacterial OTUs (Fig. 5A and B), in agreement with the clustering observed in Fig. 4A and B. For both bacterial and fungal OTUs in their ordination space, CCME fractions F3 and F4, Total Carbon (TC) and Total Nitrogen (TN) are all negatively correlated with trace nutrients (Ca, K, Na) and soil pH (Fig. 5A and B). For bacteria, CCME F3 and F4, TC and TN are strongly correlated with the centroid of section A samples and the centroid of all surface samples, whereas for fungi, they are most strongly correlated with the centroid of all surface samples (Fig. 5A and B). In both instances, these parameters are negatively correlated with the centroid for section B samples. Further, trace nutrients and soil pH are strongly correlated with the centroid for section B samples, when there is no such strong correlation for fungal OTUs of section B (Fig. 5A and B). For both bacterial and fungal ordination species, moisture is more closely associated with section A samples, while oil and grease is negatively correlated with metals (As, Cd, Co, Fe, Mn and Mo) in the bacterial OTU ordination space, and to a lesser degree in the fungal OTU ordination space (Fig. 5A and B).

## Discussion

High‐resolution sequencing of microbial taxonomic markers has facilitated spatial analysis of bacterial and fungal communities in an active hydrocarbon‐remediating landfarm. Results suggest that the sustained application of hydrocarbons and management practices at the EVRAZ landfarm have selected specialized microbial communities that are highly distinct from microbial communities in a nearby soil borrow‐pit (ERW) used as a control (Figs 1 and 3). Because of the regular application of industrial hydrocarbon waste to the farm soils along with the removal of plant life, we anticipated that the landfarm would be characterized by simple and highly refined microbial communities dominated by specialist species, but surprisingly the landfarm communities are composed of a greater diversity of OTUs than the neighbouring ERW pit soil (Figs 3 and 4). The microbial communities of the landfarm are comprised of taxonomic groups that have been previously associated with hydrocarbon‐impacted and hydrocarbon‐remediating soils. This pattern was consistent in samples collected at the beginning (June) and end (November) of the landfarm operational season, with no statistical influence of month on microbial community structure detected by the GLM (Table 2). This stability confirms the hypothesis that seasonal changes and operational activities such as tillage, fertilization and hydrocarbon application are not disruptive over time, but rather work to actively maintain the communities that have established under these management conditions.

The present study provides data that supports the expectation that landfarm maintenance practices of both tillage and frequency of waste application influence the structure and profile of resident communities. The increased loading of hydrocarbon waste and fertilization at the surface of section A is what likely selects for specific hydrocarbon‐related OTUs of *Agromyces*,* Micrococcacea* and *Terrimonas* in A relative to B and C (Fig. 4A and B). Alternatively, Section B, (where the presence of standing water and lack of deep tillage (30 cm) favours an anaerobic environment), displays a noted enrichment of several fungal OTUs, not identified to genus level (Fig. 4B). The significant influence of water level on soil physiochemical properties and microbial community structure of non‐hydrocarbon‐impacted forest and freshwater wetland soils have been previously demonstrated (Brockett *et al*., 2012; Ma *et al*., 2018). Thus, the selective presence of hydrocarbon waste, combined presence of standing water and lack of tillage at depths below 10 cm contribute to landfarm community structure.

We observed that bacterial communities are more diverse across landfarm sites and depths compared to fungal communities (Fig. 3; Table S2). Fungal genera are naturally highly heterogeneous and metabolically specialized (Karigar and Rao, 2011; Hewitt *et al*., 2016), which could explain the observation that the presence and abundance of specific fungal genera and OTUs are more greatly influenced by differences between site conditions compared to bacterial assemblages (Figs 4 and 5). RDA was used to assess the influence of these parameters on bacterial and fungal community structures, and we found that strong correlation occurs with CCME‐defined F3 and F4 fractionations to all surface samples for fungal OTUs, and with all section A samples for bacterial OTUs (Fig. 5). As F3 and F4 molecules are typically more difficult to degrade, even with increased aeration at the soil surface (Seo *et al*., 2009), it stands to reason that the frequent application of hydrocarbon at section A contributes to increased concentration of these recalcitrant products in surface soils (Fig. 2). Similarly, less effective microbial assemblages and lower aeration may explain the accumulation of F3 and F4 molecules in the section C sub‐surface soils. Thus, while complete remediation of F3 and F4 by section A microbial communities may still require extended treatment periods, RDA suggests that the differentially abundant bacterial and fungal OTUs of section A are uniquely adapted to higher concentration of recalcitrant hydrocarbons. Surprisingly, section A surface samples demonstrate increased moisture content compared with section B (Figs 2 and 5), suggesting a positive relationship between hydrocarbon waste and retention of soil moisture. This finding provides context and support for previous analysis indicating that remediation activity is optimal with increased soil moisture, in addition to aeration (Bahmani *et al*., 2018).

These results suggest that effective management practices would include removal of standing water from section B and spread section A soils to seed sites B and C, to maintain and potentially increase the efficiency of bioremediation. Furthermore, the more anaerobic deep soil B, which also contains specific hydrocarbon‐associated OTUs, if spread throughout section B, and potentially to other sections, may contribute to increased remediation activity. Ultimately, it is evident that management practices and geochemical parameters contribute to the diversity and stability of microbial community structure, and that current conditions of the operational landfarm maintained by EVRAZ supports bacterial and fungal communities capable of contributing to effective remediation of hydrocarbon waste.

## Experimental procedures

### Site details

EVRAZ Inc. North America (hereafter EVRAZ), is a leading manufacturer of tubular steel products located in Regina, Canada. EVRAZ has maintained a landfarm since 1994 for remediation of hydrocarbon spills, which includes lubricants, fuels, solvents, hydraulic fluids and antifreezes used during steel manufacturing. The landfarm is located in the Dark Brown Soil Zone of the Western Canadian Prairies. Soils here are described as dark brown, Vertisolic soils with very fine, uniform, calcareous lacustrine deposit parent material. Routine soil sampling of the landfarm indicates that toxic hydrocarbons from applied waste are degraded in the first few centimetres of soil; a success which has been attributed in some part to the landfarm maintenance programme that includes nitrogen fertilization, aeration through tilling, regular removal of standing water, occasional application of sanitary sewage treatment plant sludge and pH monitoring (Schutzman, 2014).

There are three separate management sections of the landfarm, A, B and C, which surround a waste holding area (Fig. 1). Hydrocarbon waste is continuously collected from steel plant activities and stored over the course of fall, winter and early spring months (Table 1). Sections A and C share similar soil characteristics; however, section A has historically received more waste than section C. Section B has the lowest hydrological grade, with water accumulating in this section after snowmelt or heavy periods of summer precipitation. In 2015, application of accumulated waste to sections A and C began in April and occurred periodically until late November. After the majority of standing water was removed, in early summer and fall, soils were tilled, followed by fertilization with urea in November (Table 1).

### Sample collection

Samples were collected with an AMs Frozen Soil Powered Auger Kit at two depths, ‘surface' (0–15 cm) and ‘deep' (15–30 cm), at ten sites sections (A, B, and C) of the landfarm, following tilling of the site in June 2015, with ‘surface' samples (0–15 cm) collected again in November (Table 1). For characterization of non‐impacted soils, an additional sample was taken by gloved‐hand using gloves from the top 5 cm of soil in an area ~ 500 m outside the landfarm for characterization of non‐impacted soils (ERW borrow‐pit; Fig. 1). From bagged soil samples for each depth, approximately 50 g was subsampled by gloved‐hand and processed for bacterial and fungal community analysis. Soils collected from each of the ten individual sites per landfarm section were analysed via sequencing of taxonomic gene markers as independent samples and not composited. The remainder of the June samples were composited into landfarm sections (A, B and C) at each depth and sent to Saskatchewan Research Council Environmental Analytical Laboratories (Saskatoon, SK) for routine analysis of pH, trace micronutrients (Ca, K, Na), metals (As, Cd, Co, Fe, Mn, Mo), total organic carbon, total nitrogen, phosphorus, oil and grease, and hydrocarbons: benzene, toluene, ethylbenzene, and xylene (BTEX) and CCME hydrocarbon fractions F1 – F4 (Fig. 2 and Table S1; CCME, 2008).

### Microbial community profiling

Environmental DNA was isolated from 0.25 g of homogenized soil samples using MoBio Powersoil DNA Isolation (Carlsbad, CA) according to manufacturer's instructions. Bacterial and fungal sequencing libraries were prepared according to Kozich *et al*. (2013), using primers designed for the v4 hypervariable region of the bacterial 16s rRNA gene, and primers for the fungal internal transcribed sequence (ITS‐2) region (Schoch *et al*., 2012; Gweon *et al*., 2015). Sequencing of a bacterial mock community (BEI Resources; Manassas, VA, USA) facilitated calculation of the sequencing error rate (Kozich *et al*., 2013). Sequencing was performed on the Illumina MiSeq platform for both bacterial libraries (V2; 250 bp paired‐end reads) and fungal libraries (V3; 300 bp paired‐end reads).

Bacterial sequencing data were processed using Mothur (v.1.39.5; Kozich *et al*., 2013) to remove low quality reads and perform OTU assignment at the 97% identity level, utilizing the SILVA reference bacterial database (v.123; Quast *et al*., 2013) for taxonomic identification. Fungal sequencing reads were processed for quality and analysed using the PIPITS fungal taxonomic assignment software (v.1.4.2; Gweon *et al*., 2015) with the UNITE fungal ITS reference database (v.7.0; Kõljalg *et al*., 2013).

Prior to all subsequent analysis, data were rarefied to the level of 5,307 and 11,077 sequences for bacterial and fungal samples respectively. Inverse Simpson diversity was calculated for all samples and then analysed using a GLM to determine the effect of section, depth and month of sampling as factor covariates. An interaction between landfarm section and soil depth was included in the GLM. Sample diversity is a positive real‐valued response variable with strong mean–variance relationship. To account for this, we assumed the response was conditionally distributed following a Gamma distribution with support on the positive real values. Generalized linear hypothesis tests of differences between selected treatment effects were performed using contrast coding and *P*‐values adjusted for multiple comparisons via the multcomp package (Hothorn *et al*., 2008) for r (v 3.5.1; R Core Team, 2018). Abundance of bacterial and fungal taxa from all collected samples was visualized via ClustVis (Metsalu and Vilo, 2015).

### Redundancy analysis for soil chemistry correlation

To assess relationships among geochemical parameters and community structure, RDA was performed on select parameters of the geochemical data set (Fig. 2 and Table S1), using the vegan package (v.2.41; Oksanen *et al*., 2016) for r. Bacterial and fungal OTU data were screened to remove taxa present in fewer than five samples in the data set and subsequently Hellinger transformed (Legendre and Gallagher, 2001). The month of sampling was used as a partial covariate to remove the effect of sample collection date from the data prior to analysis.

The direction and magnitude of greatest change in RDA ordination space for each geochemical variable were calculated using a least squares regression. Each geochemical variable, in turn, was used as the response variable, and the coordinates of the samples on RDA1 and RDA2 were used as covariates. The two resulting regression coefficients provide vector coordinates in the RDA space, which represent the direction of maximal change. The R^2^ statistic provides indication of the strength of the correlation between the configuration of samples in ordination space and each geochemical variable (assuming a linear relationship). Correlations were assessed statistically using a randomization test with 999 permutations.

## Conflict of interest

None declared.

## Supporting information


**Table S1.** Soil analysis results for samples collected in June.
**Table S2.** Rarefied OTU abundance table of bacterial sequences.
**Table S3.** Rarefied OTU abundance table of fungal sequences.

## References

[mbt213397-bib-0001] Adrion, A.C. , Singleton, D.R. , Nakamura, J. , Shea, D. , and Aitken, M.D. (2016) Improving polycyclic aromatic hydrocarbon biodegradation in contaminated soil through low‐level surfactant addition after conventional bioremediation. Environ Eng Sci 33: 659–670. 27678476 10.1089/ees.2016.0128 PMC5031096

[mbt213397-bib-0002] Badali, H. , Gueidan, C. , Najafzadeh, M.J. , Bonifaz, A. , Gerrits van den Ende, A.H.G. , and de Hoog, G.S. (2008) Biodiversity of the genus *Cladophialophora* . Stud Mycol 61: 175–191. 19287540 10.3114/sim.2008.61.18 PMC2610306

[mbt213397-bib-0003] Bahmani, F. , Ataei, S.A. , and Mikaili, M.A. (2018) The effect of moisture content variation on the bioremediation of hydrocarbon contaminated soils: modeling and experimental investigation. J Environ Anal Chem 5: 2.

[mbt213397-bib-0004] Bell, T.H. , El‐Din Hassan, S. , Lauron‐Moreau, A. , Al‐Otaibi, F. , Hijiri, M. , Yergeau, E. , and St‐Arnaud, M. (2014) Linkage between bacterial and fungal rhizosphere communities in hydrocarbon‐contaminated soils is related to plant phylogeny. ISME J 8: 331–343. 23985744 10.1038/ismej.2013.149 PMC3906811

[mbt213397-bib-0005] Besalatpour, A. , Hajabbasi, M.A. , Koshgoftarmanesh, A.H. , and Dorostkar, V. (2011) Land farming process effects on biochemical properties of petroleum‐contaminated soils. Soil Sed Contam 20: 234–248.

[mbt213397-bib-0006] Bourdel, G. , Roy‐Bolduc, A. , St‐Arnaud, M. , and Hijiri, M. (2016) Concentration of petroleum‐hydrocarbon contamination shapes fungal endophytic structure plant roots. Front Microbiol 7: 685. 27433155 10.3389/fmicb.2016.00685 PMC4922216

[mbt213397-bib-0007] Brockett, B.F.T. , Prescott, C.E. , and Grayston, S.J. (2012) Soil moisture is the major factor influencing microbial community structure and enzyme activities across seven biogeoclimatic zones in western. Can Soil Biol Biochem 44: 9–20.

[mbt213397-bib-0008] CCME (2008) Report: Canada wide Standard for Petroleum Hydrocarbons in Soil, 2008 URL www.ccme.ca.

[mbt213397-bib-0009] de Garcia, V. , Zalar, P. , Brizzio, S. , Gunde‐Cimermacn, N. , and van Broock, M. (2012) Cryptococcus species (*Tremellales*) from glacial biomes in the southern (Patagonia) and northern (Svalbard) hemispheres. FEMS Microbiol Ecol 82: 523–539. 22861821 10.1111/j.1574-6941.2012.01465.x

[mbt213397-bib-0010] Grabowski, A. , Tindall, B.J. , Bardin, V. , Blanchet, D. and Jeanthon, C. (2005) *Petrimonas sulfuriphila* gen. nov., sp. nov., a mesophilic fermentative bacterium isolated from a biodegraded oil reservoir. Inst J Syst Evol Microbiol 55, 1113–1121. 10.1099/ijs.0.63426-0 15879242

[mbt213397-bib-0011] Gweon, H.S. , Oliver, A. , Taylor, J. , Booth, T. , Gibbs, M. , Read, D.S. , *et al* (2015) PIPITS: and automated pipeline for analyses of fungal internal transcribed spacer sequences from the Illumina sequencing platform. Meth Ecol Evol 6: 973. 10.1111/2041-210X.12399 PMC4981123 27570615

[mbt213397-bib-0012] Harmsen, J. , Rullens, W.H. , Sims, R.C. , Rijtema, P.E. , and Zweers, A.J. (2006) Theory and application of landfarming to remediate polycyclic aromatic hydrocarbons and mineral oil‐contaminated sediments; beneficial reuse. J Environ Qual 36: 1112–1122. 10.2134/jeq2006.0163 17596619

[mbt213397-bib-0013] Hewitt, S.K. , Foster, D.S. , Dyer, P.S. , and Avery, S.V. (2016) Phenotypic heterogeneity in fungi: Importance and methodology. Fungal Biol Rev 30: 176–184.

[mbt213397-bib-0014] Hothorn, T. , Bretz, F. , and Westfall, P. (2008) Simultaneous inference in general parametric models. Biometrical J 50: 346–363. 10.1002/bimj.200810425 18481363

[mbt213397-bib-0015] Karigar, C.S. and Rao, S.S. (2011) Role of microbial enzymes in the bioremediation of pollutants: a review. Enz Res 2011: Article ID 805187. 10.4061/2011/805187 PMC3168789 21912739

[mbt213397-bib-0016] Katsivela, E. , Moore, E.R.B. , Maroukli, D. , Strömpl, C. , Pieper, D. , and Kalogerakis, N. (2005) Bacterial community dynamics during *in‐situ* bioremediation of petroleum waste sludge in landfarming sites. Biodegradation 16: 169–180. 15730027 10.1007/s10532-004-4883-y

[mbt213397-bib-0017] Khan, F.L. , Husain, T. , and Hejazi, R. (2004) An overview and analysis of site remediation technologies. J Environ Manang 71: 95–122. 10.1016/j.jenvman.2004.02.003 15135946

[mbt213397-bib-0018] Kolařík, M. , Spakowicz, D.J. , Gazis, R. , Shaw, J. , Kubátová, A. , Nováková, A. , *et al* (2017) *Biatriospora* (Ascomycota: Pleosporales) is an ecologically diverse genus including facultative marine fungi and endophytes with biotechnological potential. Plant Syst Evol 303: 35–50.

[mbt213397-bib-0019] Kõljalg, U. , Nilsson, R.H. , Abarenkov, K. , Tedersoo, L. , Taylor, A.F. , Bahram, M. , *et al* (2013) Towards a unified paradigm for sequence‐ based identification of fungi. Mol Ecol 22: 5271. 24112409 10.1111/mec.12481

[mbt213397-bib-0020] Kozich, J.J. , Westcott, S.L. , Baxter, N.T. , Highlander, S.K. , and Schloss, P.D. (2013) Development of a dual‐index sequencing strategy and curation pipeline for analyzing amplicon sequence data on the Miseq Illumina sequencing platform. Appl Environ Microbiol 79: 5112. 23793624 10.1128/AEM.01043-13 PMC3753973

[mbt213397-bib-0021] Leahy, J.G. , Tracy, K.D. , and Eley, M.H. (2003) Degradation of mixtures of aromatic and chloroaliphatic hydrocarbons by aromatic hydrocarbon‐degrading bacteria. FEMS Microbiol Ecol 43: 271–276. 19719688 10.1111/j.1574-6941.2003.tb01067.x

[mbt213397-bib-0022] Legendre, P. , and Gallagher, E.D. (2001) Ecologically meaningful transformations for ordination of species data. Oecologia 129: 271–280. 28547606 10.1007/s004420100716

[mbt213397-bib-0023] Liang, B. , Wang, L.‐Y. , Zhou, Z. , Mbadinga, S.M. , Zhou, L. , Liu, J.‐F. , *et al* (2016) Frequency of *Thermodesulfovibrio* spp. and *Anaerolineaceae* in association with *Methanoculleus* spp. in a long‐term incubation of *n*‐alkanes‐degrading methanogenic enrichment culture. Front Microbiol 7: 1431. 27695441 10.3389/fmicb.2016.01431 PMC5025540

[mbt213397-bib-0024] Ma, Y. , Li, J. , Wu, J. , Kong, Z. , Feinstein, L.M. , Ding, X. , *et al* (2018) Bacterial and fungal community composition and functional activity associated with lake wetland water level gradients. Sci Rep 8: 760. 29335587 10.1038/s41598-018-19153-z PMC5768796

[mbt213397-bib-0025] Marchand, C. , St‐Arnaud, M. , Hogland, W. , Bell, T.H. , and Hiri, M. (2017) Petroleum biodegradation capacity of bacteria and fungi isolated from petroleum‐contaminated soil. Int Biodet Biodeg 116: 48–57.

[mbt213397-bib-0026] McFarlin, M.K. , Perkins, M.J. , Field, J.A. , and Leigh, M.B. (2018) Biodegradation of crude oil and Corexit 9500 in Arctic seawater. Front Microbiol 9: 1788. 30147678 10.3389/fmicb.2018.01788 PMC6096335

[mbt213397-bib-0027] Metsalu, T. , and Vilo, J. (2015) Clustvis: a web tool for visualizing clustering of multivariate data using Principal Component Analysis and heatmap. Nucl Acids Res 43: W566–W570. 25969447 10.1093/nar/gkv468 PMC4489295

[mbt213397-bib-0028] Militon, C. , Boucher, D. , Bachelor, C. , Perchet, G. , Barra, V. , Trouquet, J. , *et al* (2010) Bacterial community changes during bioremediation of aliphatic hydrocarbon‐contaminated soil. FEMS Microbiol Ecol 74: 669–681. 21044099 10.1111/j.1574-6941.2010.00982.x

[mbt213397-bib-0029] Mohammadian, E. , Arzanlour, M. , and Babai‐Ahari, A. (2017) Diversity of culturable fungi inhabiting petroleum‐contaminated soils in Southern Iran. Ant van Leeuw 110: 903–923. 10.1007/s10482-017-0863-1 28353091

[mbt213397-bib-0030] Oksanen, J. , Blanchet, F.G. , Friendly, M. , Kindt, R. , Legendre, P. , McGlinn, D. , *et al* (2016) vegan: Community Ecology Package. R package version 2.4‐1. URL https://CRAN.R-project.org/package=vegan

[mbt213397-bib-0031] Orlygsson, J. , and Kristjansson, J.K. (2014) The Family Hydrogenophilaceae In The Prokaryotes. Rosenberg E., DeLong E.F., Lory S., Stackebrandt E., and Thompson F. (eds). Berlin, Heidelberg: Springer, pp. 859–868.

[mbt213397-bib-0032] Peng, M. , Zi, Z. , and Wang, Q. (2015) Bacterial community diversity of oil‐contaminated soil assessed by high throughput sequencing of 16S rRNA genes. Int J Environ Res Pub Health 12: 12002–12015. 26404329 10.3390/ijerph121012002 PMC4626951

[mbt213397-bib-0033] Prenafeta‐Boldu, F. , Summerbell, R. , and Sybren de Hoog, G. (2005) Fungi growing on aromatic hydrocarbons: biotechnology's unexpected encounter with biohazard? FEMS Microbiol Rev 30: 109–130. 10.1111/j.1574-6976.2005.00007.x 16438682

[mbt213397-bib-0034] Quast, C. , Pruesse, E. , Yilmaz, P. , Gerken, J. , Schweer, T. , Yarza, P. , *et al* (2013) The SILVA ribosomal RNA gene database project: improved data processing and web‐based tools. Nucl Acids Res 41: D590. 23193283 10.1093/nar/gks1219 PMC3531112

[mbt213397-bib-0035] R Core Team (2018) R: A Language and Environment for Statistical Computing. Vienna, Austria: R Foundation for Statistical Computing.

[mbt213397-bib-0036] Rhykerd, R.L. , Crews, B. , McInnes, K.J. , and Weaver, R.W. (1999) Impact of bulking agents, forced aeration, and tillage on remediation of oil‐contaminated soil. Biores Technol 67: 279–285.

[mbt213397-bib-0037] Schoch, C.L. , Seifert, K.A. , Huhndorf, S. , Robert, V. , Spouge, J.L. , Levesque, C.A. , *et al* (2012) Nuclear ribosomal internal transcribed spacer (ITS) region as a universal DNA barcode marker for Fungi. PNAS 109: 6241. 22454494 10.1073/pnas.1117018109 PMC3341068

[mbt213397-bib-0038] Schutzman, R. (2014) EVRAZ Inc. NA Canada: Landfarm Management Guide, 2nd edn EVRAZ Inc. NA Canada: Regina, Canada.

[mbt213397-bib-0039] Seo, J.S. , Keum, Y.S. , and Li, Q.X. (2009) Bacterial degradation of aromatic compounds. Int J Environ Res Pub Health 6: 278–309. 19440284 10.3390/ijerph6010278 PMC2672333

[mbt213397-bib-0041] Straube, W.L. , Nestler, C.C. , Hansen, L.D. , Ringleberg, D. , Pritchard, P.H. , and Jones‐Meehan, J. (2003) Remediation of polyaromatic hydrocarbons (PAHs) through landfarming with biostimulation and bioaugmentation. Eng Life Sci 23: 170–196.

[mbt213397-bib-0042] Wang, S.Y. , Kuo, Y.C. , Hong, A. , Chang, Y.M. , and Kao, C.M. (2016) Bioremediation of diesel and lubricant oil‐contaminated soils using enhanced land farming system. Chemosphere 164: 558–567. 27627466 10.1016/j.chemosphere.2016.08.128

[mbt213397-bib-0043] Zhang, D.‐C. , Schumann, P. , Liu, H.‐C. , Xin, Y.‐H. , Zhou, Y.‐G. , Schinner, F. , and Margesin, R. (2010) *Agromyces bauzanensis* sp. nov., isolated from soil. Int J Syst Evol Microbiol 60: 2341–2345. 19933590 10.1099/ijs.0.017186-0

[mbt213397-bib-0044] Zhang, B. , Xu, X. , and Zhu, L. (2017) Structure and function of the microbial consortia of activated sludge in typical municipal wastewater treatment plants in winter. Sci Rep 7: 17930. 29263391 10.1038/s41598-017-17743-x PMC5738398

